# Identification of new markers for the *Schistosoma mansoni* vitelline lineage^☆^

**DOI:** 10.1016/j.ijpara.2016.03.004

**Published:** 2016-06

**Authors:** Jipeng Wang, James J. Collins

**Affiliations:** Department of Pharmacology, UT Southwestern Medical Center, Dallas, TX 75390, USA

**Keywords:** *Schistosoma*, Reproduciton, Stem cell, Vitellaria

## Abstract

Schistosomes cause significant morbidity and mortality in millions of the world’s poorest people. While parasite egg-induced inflammation is the primary driver of host pathology, relatively little is known at the molecular level about the organ systems that participate in schistosome egg production (i.e., testes, ovaries and vitellaria). Here we use transcriptional profiling and in situ hybridization to characterise the vitellarium of *Schistosoma mansoni*. We uncovered several previously uncharacterised vitellaria-specific factors and defined molecular markers for various stages in the vitellocyte differentiation process. These data provide the framework for future in-depth molecular studies exploring the biology of this important parasite organ.

## Highlights

• Transcriptional profiling identifies candidate factors associated with the schistosome vitellarium.
• In situ hybridization confirms many new markers of this tissue.
• New cell type-specific markers for various stages of vitellocyte development are reported.

## Graphical abstract

**Figure:**
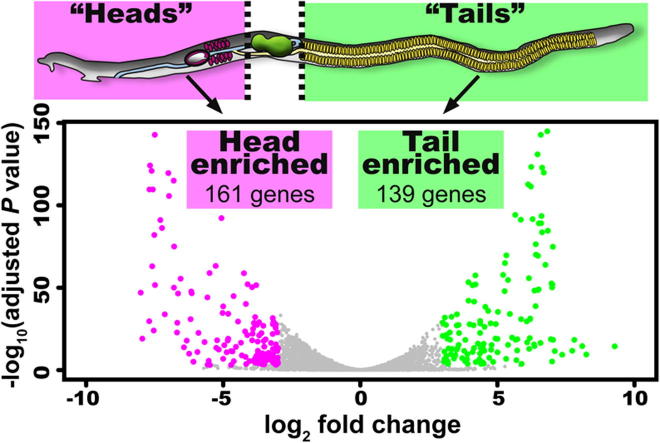

A schistosome worm pair can lay an egg every 1–5 min in vivo (Basch, 1991). Similar to all neoophoran flatworms (i.e., all parasitic flatworms and a subset of derived free-living worms (Martin-Duran and Egger, 2012, Collins and Newmark, 2013, Laumer et al., 2015), schistosomes produce ectolecithal (i.e., yolk on the outside) eggs consisting of an oocyte surrounded by specialised “yolk” cells known as vitellocytes (Gönnert, 1955, Basch, 1991, Shinn, 1993, Collins et al., 2011). Vitellocytes provide both nutrition for the developing zygote and constituents essential for the construction of the polyphenolic egg shell (Basch, 1991, Shinn, 1993). Vitellocytes are produced by a specialised organ, the vitellarium, that occupies the posterior two-thirds of the female schistosome body (Fig. 1A). This tissue is composed of a network of thousands of follicles (vitellaria) in which specialised stem cells, called S1 cells, differentiate to ultimately produce mature vitellocytes (S4 cells) (Fig. 1A) (Erasmus, 1975). These mature vitellocytes are fed anteriorly though the vitelline duct and are joined with fertilised oocytes in the ootype where the mature egg is formed.

In both a biological and therapeutic sense, the vitellarium is an intriguing organ. From a biological perspective, vitellaria and ectolecithal eggs are evolutionarily-derived features absent from basal flatworms, where the oocytes are the exclusive source of yolk (Martin-Duran and Egger, 2012). How did the production of ectolecithal eggs evolve? What advantage does ectolecithal egg production afford these worms? What is the developmental relationship between the vitellaria and the female germ line? From a therapeutic viewpoint, the vitellarium represents an attractive therapeutic target since egg production promotes both disease transmission and host pathology (Pearce and MacDonald, 2002). Moreover, since vitellaria are a flatworm-specific innovation, it is possible that parasite-specific therapeutics targeting this organ could be developed. Unfortunately, no systematic molecular characterisation of the schistosome vitellarium has been reported to date. Here we describe our initial efforts to characterise this organ at a molecular level.

Recently, important tools to study the schistosome vitellarium, including tissue isolation (Hahnel et al., 2013) and Fluorescence Activated Cell Sorting (Lu et al., 2015), have been reported. While potentially powerful, these approaches are technically demanding, requiring a large number of parasites to generate sufficient material for standard transcriptional profiling. Therefore, we took advantage of the fact that the posterior of the female schistosome is almost entirely vitellaria (Fig. 1A). Since tissues such as the intestine, tegument and protonephridia are evenly distributed in the worm, we reasoned that comparing the transcriptional profiles of “heads” versus “tails” would allow us to identify transcripts enriched in the vitellarium. To perform these experiments, we separated adult male and female *Schistosoma mansoni* (recovered from mice 7–8 weeks p.i.) as previously described (Collins et al., 2013). Using a sharpened tungsten needle, we amputated females both anterior to and posterior to the ovary (Fig. 1B). We then prepared RNA from ∼100 anterior or posterior worm fragments using Trizol (Invitrogen, Carlsbad, CA, USA) and DNase treatment (DNA-free RNA Kit, Zymo Research, Irvine, CA, USA). Three independent rounds of RNA extraction were performed and samples were submitted to the University of Texas Southwestern Medical Center Genomics Core Facility, USA, for RNAseq library preparation (TruSeq Stranded RNA LT Kit, Illumina, San Diego, CA, USA) and sequencing (Illumina HiSeq2500, San Diego, CA, USA). Reads were mapped to the schistosome genome (Berriman et al., 2009, Protasio et al., 2012) with STAR (v 2.5.0b) (Dobin et al., 2013). The genome sequence and gff3 files were downloaded from Wormbase Parasite (v4) (Howe et al., 2016). Differential gene expression analysis was performed using DESeq v1.22 (Anders and Huber, 2010) (Supplementary Table S1).

Our RNAseq analysis identified 161 head-enriched (log_2_ fold change ⩽ −3, *P* < 0.001) and 139 tail-enriched (log_2_ fold change ⩾ 3, *P* < 0.001) transcripts (Fig. 1B, Supplementary Table S2). Our tail-enriched dataset included many of the known vitellaria-specific factors including two tyrosinases (Smp_050270; Smp_013540) (Fitzpatrick et al., 2007), a superoxide dismutase (Smp_095980) (Cogswell et al., 2011), *female specific protein 800* (Smp_000290) (Reis et al., 1989), *p48* (Smp_014610) (Chen et al., 1992), and *p14* (Smp_131110) (Bobek et al., 1988) (Supplementary Table S3), confirming the utility of our approach. Additionally, the tail-enriched data set included nine of the 11 genes in the *S. mansoni* genome annotated as containing a “Trematode Egg Shell” domain (Pfam ID: PF08034) (Supplementary Table S3). These proteins and others are thought to be the major structural components of the egg shell in schistosomes and other trematodes (Ebersberger et al., 2005). Given that the vitellarium is a flatworm-specific organ, it is not surprising that a large fraction of the genes in this dataset are annotated as hypothetical proteins.

To further examine our RNAseq dataset, we cloned cDNA fragments for 54 genes (42 tail-enriched and 12 head-enriched; Supplementary Table S4) as described previously (Collins et al., 2010). Some of the genes selected were previously shown to be expressed in the vitellaria (e.g., *tyrosinase 1*); the remainder were selected essentially at random. Using these cloned cDNAs, we generated antisense riboprobes and performed a whole-mount in situ hybridization (WISH) screen of female worms (Collins et al., 2013). Of the 42 tail-enriched genes screened, we found mRNAs for 32 genes that were either exclusive to or highly enriched in the vitellaria (Fig. 2A) (Supplementary Table S4). The remaining 10 tail-enriched transcripts were either not detected, expressed at low levels with unclear expression patterns, or ubiquitously expressed (Supplementary Table S4). Nevertheless, these data confirm the utility of our approach to identify vitellaria-expressed transcripts. In addition to confirming the expression of known vitellaria-specific factors (e.g., *superoxide dismutase* and both *tyrosinases*)*,* we identified several novel markers for the schistosome vitellarium (Fig. 2A) (Supplementary Table S4). These included genes predicted to encode enzymes (e.g., a serine hydroxymethyltransferase, a peptidase and a glutathione peroxidase), an amino acid transporter, eggshell proteins (*tes1* and *tes2*), a homologue of the *Drosophila* nuclear hormone receptor Ecdysone-Induced protein 78c (*e78*), and several hypothetical proteins. Nanos proteins are RNA-binding proteins that play essential roles in germ line development throughout metazoa (Seydoux and Braun, 2006). In free-living flatworms, *nanos* expression is nearly exclusive to the presumptive stem cells in the male and female germ line (Wang et al., 2010, Wang et al., 2007). Consistent with observations in free-living flatworms, we observed *nanos-1* expression in the anterior portion of the ovary where the female germ line stem cells, or oogonia, are thought to exist (Fig. 2A) (Nollen et al., 1976). Interestingly, we also detected expression of *nanos-1* mRNA in small patches of cells scattered throughout the vitellaria (Fig. 2A).

Furthermore, we examined the expression of genes from our head-enriched dataset by whole mount in situ hybridization. Consistent with a previous study that compared gene expression in the head versus tail of male *S. mansoni* (Wilson et al., 2015)*,* we identified several Venom Allergen proteins (VALs) and Mini Exon Genes (MEGs) that were expressed in the worm’s oesophageal gland (Fig. 2B) (Supplementary Table S4). We also observed an mRNA encoding a Wnt protein that was expressed along the lateral margins of female parasites (Fig. 2B). This expression started just behind the head of the worm and extended posteriorly approximately halfway down the length of the worm (Fig. 2B). Intriguingly, this Wnt protein appears to be orthologous to the protein encoded by the planarian *wntP-2* gene. This planarian gene is expressed in a gradient along the planarian posterior and plays a role in re-establishing polarity during regeneration (Petersen and Reddien, 2009). Thus, this schistosome Wnt protein could play a role in establishing and maintaining axial polarity during parasite growth and homeostatic tissue renewal. Our analysis also identified new markers for two components of the female reproductive tract: Mehlis’ gland and the ootype. The function of Mehlis’ gland is unclear but it has been hypothesised to perform a variety of functions including providing lubrication for the reproductive tract, activating sperm and providing constituents important for egg shell formation (Smyth and Halton, 1983). The ootype sits just anterior to Mehlis’ gland and is responsible for eggshell formation (Fig. 1A). We observed cells in the proximity of Mehlis’ gland strongly expressing a homologue of the Ca^2^^+^ binding protein Reticulocalbin 2 and cells in the ootype expressing a schistosome-specific hypothetical protein (*Smp_124750*) (Fig. 2B). We also observed expression of a Jagged homologue (*jag1*) in the region of Mehlis’ gland (Fig. 2B). Jagged proteins are cell surface ligands that activate Notch signalling in adjacent cells (Kopan and Ilagan, 2009). With the expression of this Jagged molecule in Mehlis’ gland, it is possible that the Notch pathway becomes activated in cells (i.e., vitellocytes, oocytes or sperm) as they transit near Mehlis’ gland on their way through the oviduct. Such activation could be critical to initiating processes such as eggshell formation (in the case of vitellocytes or oocytes) or fertilisation (in the case of sperm). Examination of other Notch signalling components could provide support for this hypothesis.

Upon closer examination we noted that many tail-enriched genes were expressed in distinct cell types within the vitellaria. For instance, the expression of some genes was restricted to the vitellaria (e.g., *tyrosinase 1*), whereas others were expressed both in the vitellaria and vitelline duct (*Smp_000400*) (Fig. 2A). These two groups of expression could be divided even further, since some genes appeared to be expressed in subsets of cells within the vitellaria (e.g., *cryβγ-1* and *nanos-1*). Since the cells within the vitelline duct represent the mature vitellocytes (S4 cells), we reasoned that genes whose expression is highly enriched in the vitellaria and not the vitelline duct might represent factors expressed at earlier stages of vitellogenesis. To explore this idea further, we conducted fluorescence in situ hybridization (FISH) experiments with Fluorescein- and Digoxigenin-labelled riboprobes as described previously (Collins et al., 2013). Detection of transcripts was performed using peroxidase-conjugated antibodies and sequential rounds of Tyramide Signal Amplification (TSA); residual peroxidase activity between rounds of TSA was quenched with 100 mM Sodium Azide in TNT (0.1 M Tris pH 7.5, 0.15 M NaCl and 0.1% Tween-20) for 45 min (King and Newmark, 2013). Following TSA detection, animals were incubated for 1 h in 10 mM CuSO_4_ and 50 mM NH_4_CH_3_CO_2_ pH 5.0 to quench autofluorescence within the vitellaria (King and Newmark, 2013).

We performed FISH with riboprobes for *nanos-1*, *tes1*, *cryβγ-1* or *Smp_000400* with the proliferative cell marker *Histone H2B*. Since *Histone H2B* expression is restricted to proliferative cells in the schistosome soma and germ line (Collins et al., 2013), we reasoned that it would be expressed in the proliferative S1 stem cells and perhaps in their early differentiation progeny within the vitellaria. Consistent with this hypothesis, *Histone H2B* expression was limited to small cells that incorporated the thymidine analogue 5-ethynyl-2′-deoxyuridine (EdU) and did not possess the cytoplasmic granularity characteristic of more differentiated cells in the vitellarium (Fig. 3A and B). By double FISH, we found that nearly all *Histone H2B^+^* cells were also positive for *nanos-1*, indicating that *nanos-1* is a marker for the schistosome S1 cells (Fig. 3B). Given the highly conserved role of Nanos proteins in germ cell development, this observation provides molecular evidence that the vitellocyte lineage may be evolutionarily derived from the germ line. In contrast to *nanos*-1, we did not observe co-expression of *tes1*, *cryβγ-1*, or *Smp_000400* with *Histone H2B,* suggesting that these genes are not expressed in the S1 cells (Fig. 3B). Consistent with this idea, these genes were all expressed in cells possessing a highly granular cytoplasm (Fig. 3B). Similar to our WISH results, these genes all had distinct patterns of expression: *tes1* was highly expressed in the majority of cells within the vitellaria and not highly expressed in the cells within the vitelline duct; *cryβγ-1* was highly expressed in cells within the vitelline duct and a subset of cells in the vitellaria located proximal to the vitelline duct; and *Smp_000400* appeared to be broadly expressed in both the vitellaria and cells within the vitelline duct. Based on these patterns of expression we suggest a model for vitelline cell development in which *nanos-1^+^/Histone H2B^+^* S1 stem cells differentiate to *tes1*^+^/*Smp_000400*^+^ cells before becoming mature S4 vitellocytes that express high levels of both *cryβγ-1* and *Smp_000400*. Whether the *tes1*^+^/*Smp_000400*^+^ cells represent the S2–S3 cells defined in the classic literature using electron microscopy (Fig. 1A) (Erasmus, 1975) cannot be determined at this time. Future studies utilising these markers in concert with EdU pulse-chase approaches (Collins et al., 2013) will provide unambiguous support for our proposed model of vitellocyte differentiation.

Here we provide an initial characterisation of the schistosome vitellarium. In sum, we validated several novel factors expressed in this organ system and identified new cell type-specific markers for S1 cells and their differentiation progeny. Combination of the techniques outlined here with the emerging approaches to isolate distinct vitelline cell populations (Lu et al., 2015), should allow for a higher resolution description of the developmental events culminating in vitellocyte production. Furthermore, since vitellaria are a flatworm-specific evolutionary innovation, we suspect these data will provide a starting point for the molecular characterisation of this organ in other flatworms. Indeed, identification of molecules essential for vitellocyte production in more experimentally tractable flatworm models (e.g., planarians) could provide a path towards understanding the biology of this fascinating organ while simultaneously identifying therapeutic targets against schistosomes and other parasitic flatworms.

## Figures and Tables

**Fig. 1:**
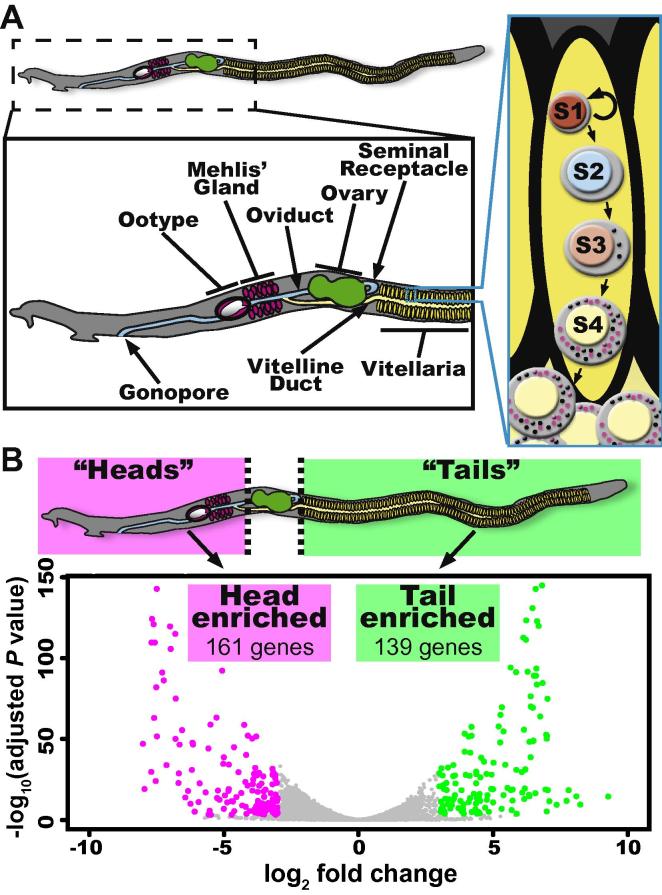
Identification of genes associated with the schistosome vitellarium. (A) Cartoon of the schistosome reproductive system and stages of vitellocyte development (inset boxed in cyan). Within the vitellaria, S1 cells proliferate and differentiate to ultimately generate S4 vitellocytes that are rich in lipid (magenta circles) and vitelline (black circles) droplets. These mature vitellocytes pass to the vitelline duct and travel anteriorly to the ootype. (B) Cartoon depicts the strategy to identify vitellaria associated transcripts. To identify genes associated with the vitellaria, RNA from amputated female heads and tails were subjected to RNAseq. The Volcano Plot depicts the 161 head-enriched (magenta dots) and 139 tail-enriched (green dots) transcripts. Genes not reaching our fold-change and significance thresholds are depicted as grey dots.

**Fig. 2:**
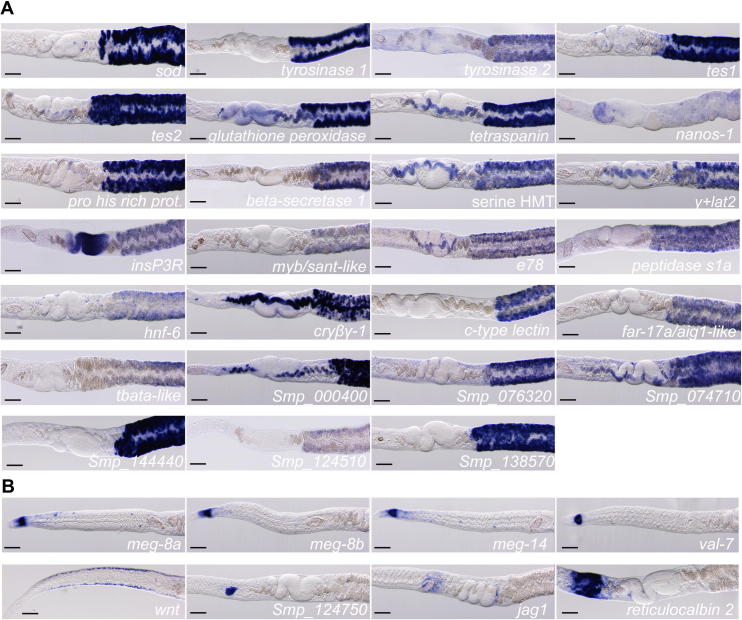
Whole mount in situ hybridization for head- and tail-enriched schistosome transcripts. Expression of (A) tail-enriched or (B) head-enriched transcripts. Gene names are listed below each image. Genes without clear homologues of known function or recognisable domains are listed with their Smp number. Details of genes examined are provided in Supplementary Table S4. Over 75% of tail-enriched transcripts examined by whole mount in situ hybridization were expressed in the vitellaria, whereas the head-enriched transcripts were detected in the oesophageal glands, the ootype, Mehlis’ Gland, and in cells within the parenchyma. Images were captured using a Zeiss AxioZoom.V16 equipped with a transmitted light base and a Zeiss AxioCam 105 Color camera. Anterior is to the left. Scale bars = 100 μm.

**Fig. 3:**
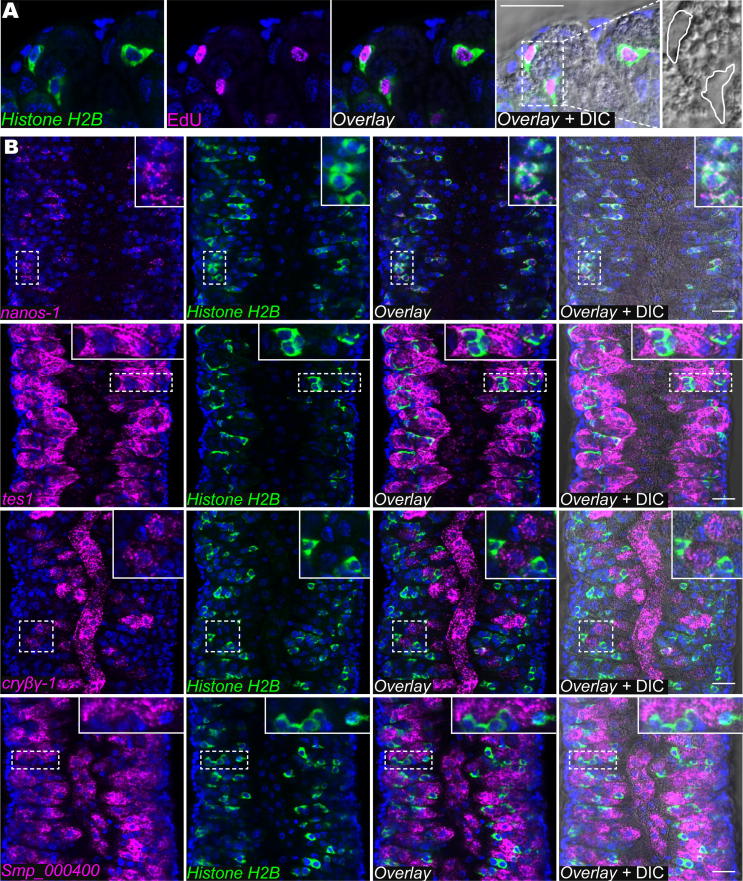
Fluorescence in situ hybridization identifies markers for unique cell populations within the schistosome vitellaria. (A) 5-ethynyl-2′-deoxyuridine labelling and FISH for *Histone H2B*. Parasites were treated for 4 h with the thymidine analogue 5-ethynyl-2′-deoxyuridine, fixed and processed for FISH as previously described (Collins et al., 2013). 5-ethynyl-2′-deoxyuridine incorporation following a 4 h pulse appeared to be exclusive for *Histone H2B^+^* cells in the vitellaria, indicating that these cells are the proliferative cells of the vitellaria and are likely to represent S1 cells. Differential Interference Contrast imaging (left) showed that these cells lack the granularity characteristic of differentiated vitelline cells, consistent with the idea that these cells are undifferentiated S1 cells. (B) Double fluorescence in situ hybridization for *Histone H2B* with *nanos-1*, *tes1*, *cryβγ-1*, or *Smp_000400*. *nanos-1* expression was restricted to *Histone H2B^+^* cells indicating that *nanos-1* is likely a marker for S1 cells. *tes1*, *cryβγ-1* and *Smp_000400* are not expressed in *Histone H2B^+^* S1 cells; rather, their expression was observed in distinct sets of more differentiated vitelline cells. *tes1* expression was highest in cells within the vitellaria and lower in the most differentiated vitelline cells within the vitelline duct. *cryβγ-1* was expressed at high levels in cells within the vitelline duct and in cells adjacent to the duct. *Smp_000400* appeared to be ubiquitously expressed in all cells of the vitelline lineage with the exception of the S1 cells. Thus, we speculate that cells expressing high levels of *tes1* and *Smp_000400* are likely to represent an intermediate step in the differentiation process of mature vitellocytes that express *cryβγ-1* and *Smp_000400*. Insets show magnified views of boxed regions. In all images, nuclei are in blue. Images were acquired using an Zeiss LSM700 laser scanning confocal microscope with either a Plan-Apochromat 63×/1.4 Oil DIC or EC Plan-Neofluar 40×/1.30 Oil DIC objective lens. Scale bars = 20 μm.

## Appendix A Supplementary data

Supplementary Table S1 Gene expression data for all schistosome genes.

Supplementary Table S2 Gene expression data for schistosome head-enriched transcripts (adj. *P* value < 0.001, log 2 Fold Change > −3).

Supplementary Table S3 Gene expression data for schistosome tail-enriched transcripts (adj. *P* value < 0.001, log 2 Fold Change < 3).

Supplementary Table S4 Details of schistosome genes examined in this study.
